# Application of *Lactobacillus acidophilus* (LA 5) strain in fruit-based ice cream

**DOI:** 10.1002/fsn3.66

**Published:** 2013-10-16

**Authors:** Suraji A Senanayake, Sirimali Fernando, Arthur Bamunuarachchi, Mariam Arsekularatne

**Affiliations:** 1 Department of Food Science and Technology, Faculty of Applied Sciences, University of Sri JayewardenepuraGangodawila, Nugegoda, Sri Lanka; 2 National Science FoundationColombo, Sri Lanka; 3 Ceylon Cold StoresRanala, Sri Lanka

**Keywords:** Ice cream, *Lactobacillus acidophilus*, probiotic

## Abstract

A study was performed to apply a probiotic strain into fermented ice cream mix with suitable fruit bases to develop a value-added product with a substantial level of viable organisms for a sufficient shelf life. Pure direct vat strain culture of *Lactobacillus acidophilus* (LA 5) in freeze-dried form was inoculated into a mixture of ice cream, frozen, and the number of viable organisms during frozen storage for a period of time was enumerated, using turbidity measurements with a spectrophotometer. An ice cream sample prepared without the probiotic culture was compared with the test sample for quality, by testing the basic quality parameters for ice cream. Results show a reduction in the over run of the probiotic ice cream compared to the nonprobiotic ice cream. Significantly high level (*P* < 0.05) of total solids (42%), proteins (16.5%), and titratable acidity (2.2%) was observed in the test sample compared to the nonprobiotic ice cream. Significantly low pH level in the probiotic sample may be due to the lactic acid produced by the probiotic culture. No significant difference (*P* > 0.05) in the fat content in the two types of ice cream was observed. A significantly low level (*P* < 0.05) of melting in the probiotic one may have resulted from less over run, than the nonprobiotic sample. Rapid reduction in the viable cells during frozen storage occurred at −18°C and gradual adaptation occurred over the first 4 weeks. At the 10th week, 1.0 × 10^7^ numbers of viable organisms were present in 1 g of the probiotic ice cream. Results show the presence of a sufficient number of viable organisms in the product for the 10-week period, which would be beneficial to consumers.

## Introduction

Incorporation of probiotic organisms into dairy food items to increase the nutritional status with the added therapeutic characteristics is practiced worldwide. Yogurts, milk, frozen yogurts, and ice cream are enriched by adding probiotic organisms in countries like Japan, USA, Australia, and Europe. Probiotics are generally mono or mixed cultures of live microorganisms which form the major component of the gut flora (e.g., lactobacilli, bifidobacteria). Probiotics, when ingested beneficially, affect the host by replenishing the depleted gut microflora, which may have occurred due to the use of antibiotics, illness and stress, travel or lifestyle changes, and also by improving the properties of the indigenous microflora of the host. Microbial strains for probiotic use must be representative of microorganisms that are generally recognized as safe microbes. The strain used in this study (*Lactobacillus acidophilus*) is commonly used around the world in food products. Various criteria for the selection of probiotic strains were proposed based on safety, efficacy, and technological production (Hoier 1992; Hull et al. 1992; Nemcova et al. 1999).

Lactobacilli are Gram-positive, lactic acid-producing bacteria that constitute a major part of the normal intestinal microflora in humans and animals and play an important role in resistance to colonization against exogenous, potentially pathogenic organisms. The lactic acid produced due to fermentation of the probiotic organisms reduces the pH of the intestine and thereby creates an environment which is not conducible for the growth of pathogenic intestinal organisms that prefer an alkaline growth environment (Vondruskova et al. 2010). Different probiotic strains will bring about varying effects, and strain-specific patterns are valuable for epidemiological studies in which bacteria isolated from patients and possible sources of infection (e.g., food, supplements) are compared to establish causality (Rautio et al. 2000).

Ice cream is a delicious, wholesome, and nutritious frozen dairy product, which is widely consumed in different parts of the world. The growing interest of consumers toward therapeutic products has led to the incorporation of probiotic cultures in ice cream, thus resulting in dietetic ice creams. The aim of this work was to apply a probiotic strain to ice cream mix to ferment with suitable fruit bases and come up with a value-added product with a substantial level of viable organisms for a sufficient shelf life.

## Materials

Pure direct vat strain culture of *L. acidophilus* (ATCC 4356) was obtained from J.L. Morison, Sons & Jones (Ceylon) Ltd, Colombo, in freeze-dried form and the samples were kept at −18°C. Fresh milk was obtained from National Livestock Development Board (NLDB) farms, Sri Lanka, for the preparation of the ice cream mix.

### Methodology

#### Preparation of the ice cream mix

An ice cream mix consisting of 37.4% of total solids (TS), 10% of fat, 11% of milk solid nonfat, 16% of sugar, 0.4% of stabilizer–emulsifier mix, and 63.6% of water was formulated. The required quantities of the ingredients for 900 mL of ice cream mix (NLDB fresh milk, margarine, skim milk powder, and drycoid as the stabilizer–emulsifier) were weighed, mixed, and heated for 50°C and the mixture was homogenized using a laboratory-scale blender for 5 min. The homogenized mixture was pasteurized at 90°C for 15 min and 50% of the prepared ice cream mix was kept at 4°C and aged for 18 h.

#### Preparation of yogurt mix

*Lactobacillus acidophilus* culture (0.1 g) was transferred into unaged ice cream mix and mixed thoroughly under sterile conditions. The mixture was incubated at 43°C for 3 h and refrigerated at 6°C to prevent further fermentation.

#### Preparation of fruit puree

Fresh wood apples were cleaned, cracked, and seeds were removed. They were mixed with a 1:1 ratio of potable water and liquidized. The mixture was pasteurized at 63°C for 30 min and refrigerated at 6°C.

#### Freezing of ice cream mix

Ice cream and yogurt mix (50% each), together with 100 mL of wood apple puree making the total volume of 1 L, was transferred into a batch freezer and air was incorporated until the volume of the total mix reached nearly the double of its initial volume. The frozen mixture was transferred into sterile containers and refrigerated at −18°C to harden the product.

An ice cream was prepared as the control with 900 mL of ice cream mix and 100 mL of fruit puree without the addition of *L. acidophilus*.

### General quality analysis of ice cream

Melting rate was determined as the average time taken to drip a scoopful of ice cream (melting rate = 1/time [min]). Over run was calculated as the weight of the ice cream mix before whipping (*X*_1_) and after whipping (*X*_2_) (% over run = *X*_2_ − *X*_1_**/***X*_1_ × 100). TS, fat, and protein contents were analyzed using Association of Official Analytical Chemistry (AOAC) (1980) (16. 287, 42. 212) methods. pH was measured using a digital pH meter (Hanna, Bangalore, India) and titratable acidity was determined as the percentage of lactic acid by titrating with 0.1 mol/L NaOH and phenolphthalein indicator.

### Enumeration of viable bacteria in the ice cream with time using spectrophotometer

A dilution series was prepared using 1 mL of the control ice cream in sterile water. Absorbance was measured at 600 nm using a spectrophotometer (UVmini – 1240; Shimadzu, Tokyo, Japan) (assumption = the absorbance ∞ turbidity and the turbidity increase due to bacterial proliferation). The dilutions were selected in the absorbance range 0.2–0.7 and the particular samples were cultured in nutrient agar using the pour plate method.

The number of organisms was measured by adding molten agar (pour plate method). One microliter of each dilution (10^−2^–10^−6^) was added into sterile petri dishes and 15 mL of nutrient agar was poured into each dish. The petri dishes were mixed well and incubated at 37°C for 24 h and the colonies were counted between 15 and 300 and the number of colony-forming units (CFU) in 1 mL of dilution was determined (CFU = no. of colonies × dilution factor).

A graph was plotted between the number of CFU in 1 mL dilution versus absorbance. Samples were taken from the prepared ice cream within different time periods (up to 10 weeks) while in frozen storage (−18°C) and the absorbance of prepared dilutions was measured (selected the dilutions in the 0.2–0.7 range). Using the standard graph, the number of organisms in the sample was determined.

Total plate count of microbes using nutrient agar was carried out in probiotic and nonprobiotic ice creams when the products were frozen after production using the bacterial enumeration method (pour plate technique).

### Statistical analysis

Samples were triplicated and results were compared by analysis of variance (ANOVA) using MINITAB ver 14 (Minitab Inc., State College, PA).

## Results and Discussion

Results of the basic ice cream tests show a reduction in the over run of the probiotic-added ice cream compared to the nonprobiotic ice cream (Table 1). TS and protein content in probiotic ice cream are significantly higher (*P* < 0.05) than in the nonprobiotic ice cream and the significantly high level of titratable acidity and the low pH level in the test sample are probably due to the lactic acid produced by the probiotic culture. There is no significant difference (*P* > 0.05) in the fat content of the two types of ice creams and a comparatively low level of melting was observed in the probiotic than in the nonprobiotic sample. The low level of melting may have resulted from the foam stability arising due to the low pH, coupled with the higher level of TS in the probiotic product. The amount of air also determines the melting rate and it is evident that low over run compared to nonprobiotic product may have been associated with the low melting rate of the probiotic ice cream.

**Table 1:** Comparison of basic quality parameters of probiotic and nonprobiotic ice creams

Quality parameter	Probiotic	Nonprobiotic
Over run (%)	63 ± 0.4^b^	75 ± 0.2^a^
Total solids (%)	42 ± 0.1^c^	38 ± 0.1^d^
Fat content (%)	11.2 ± 0.1^e^	10.5 ± 0.6^e^
Protein level (%)	16.5 ± 0.2^f^	14.2 ± 0.2^g^
pH level	5.8 ± 0.4^h^	6.3 ± 0.1^i^
Titratable acidity (%)	2.2 ± 0.4^j^	1.5 ± 0.1^k^
Melting rate (min^−1^)	23 ± 0.2^l^	15 ± 0.1^m^

Data represent the mean of three replicates. Values followed by different superscripts in each row are significantly different at the *P* < 0.05 level.

**Figure 1:**
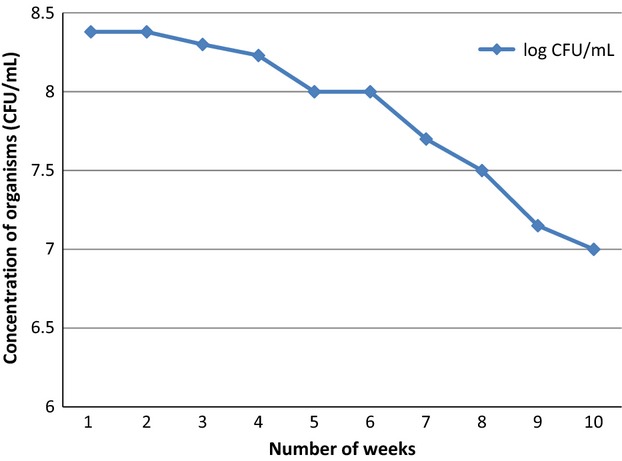
Survival of probiotic organisms with storage time.

Total plate counts for the presence of microorganisms in 1 g of probiotic and nonprobiotic ice creams are ∼1 × 10^12^ and 1 × 10^2^. From this observation, we can assume that the excess number of 1 × 10^10^ organisms in the test sample is due to the probiotic culture. Shelf life studies after the first week of frozen storage indicate a twice than 10-fold reduction in the initial number of organisms (Fig. 1).

The rapid reduction in viable cells during frozen storage may be due to the inability of the organisms to adapt to the rather low level of temperature at once (−18°C). The adaptability of the remaining organisms to the existing environment is shown in the first 2 weeks. A 10-fold reduction in viable organisms was observed between 6 and 10 weeks of frozen storage. A gradual decrease in the number of organisms can be seen over the 10-week period and at the end of the 10th week, 1.0 × 10^7^ numbers of viable organisms are present in 1 g of probiotic ice cream. In order to obtain the benefits of consuming a probios-containing product, 1 g of the product should contain 10^6^ numbers of viable organisms (Chr. Hansen, http://www.chr-Hansen.com). Therefore, the test sample can cover this requirement for more than 10 weeks. More viable organisms can ensure better survival of probiotics in the gut and be beneficial consumers who use the product on a daily basis over a period of time. Depending on the individual, the required quantity and time required for successful colonization in the gut vary. It is suggested to feed the product into selected groups of individuals for a period of time followed by enumeration of organisms in the stool samples for a more conclusive study.

## Conclusion

Probiotic ice cream shows a reduction in the over run, high level of TS and protein contents than the nonprobiotic ice cream. Increased titratable acidity and reduced pH together with low over run resulted in a low melting rate in the LA 5 direct vat strain culture-added ice cream. The number of organisms was reduced significantly in the initial stage of frozen storage and gradual reduction occurred over the tested period of time while a considerable number of organisms survived and provided beneficial effects.
